# Supplementary material for: The Impact of Age on Mortality in Chronic Haemodialysis Population with COVID-19
Source: J Clin Med. 2021 Jul 7;10(14):3022. doi: 10.3390/jcm10143022 (PMC8304096; doi:10.3390/jcm10143022)
Supplement: Supplementary file 1 [file jcm-10-03022-s001.zip › jcm-1254358-supplementary.pdf]

Journal of
Clinical Medicine

COVID-19 Impact on Elderly Hemodialysis Population

Index

Supplementary tables

Table S1 - Univariate Cox regression for mortality risk factors in non-elderly patients on haemodialysis with COVID-19. 2

Table S2 - Treatments received by elderly patients on hemodialysis with COVID-19 and univariate Cox regression analysis. 3

Table S3 - Hospitalization and mortality in Spanish general population and hemodialysis patients with COVID-19 infection (Frequencies shown in Figure 3). 4

Figure S1 - ROC curve depicting age as a predictor of mortality in elderly haemodialysis patients affected by COVID-19. 5

Members of the Spanish COVID-19 CKD Working Group 6

References 8

SUPPLEMENTARY TABLES

Table S1. - Univariate Cox regression for mortality risk factors in non-elderly patients on haemodialysis with COVID-19. Values expressed in median and interquartile range (IQR), mean ± SD or n (%). HR: Hazard ratio and 95% confidence interval for the ratio. KRT: Kidney Replacement Therapy. ICU: Intensive Care Unit. NA: Not Assessed. [a]Included cough, rhinorrhea and sputum production. [b]Included nausea, vomiting and diarrhea. [c]HR estimated for 10-years age increase. [d]HR estimated for one year of haemodialysis. [e]HR estimated for 5-days of hospitalization.

	Total (n = 254)	Survivors (n = 214)	Non-survivors (n = 40)	HR (95% CI)	*p* value
**Mean follow-up time (days)**	20 (IQR: 13-30)	22 (IQR: 14-34)	11 (IQR: 6-18.5)	NA	NA
**Age**	53.5 ± 10.7	53.1 ± 10.7	56.0 ± 10.8	1.36 (0.94 - 1.97)[c]	0.100
**Male sex**	165 (65.0%)	138 (64.5%)	27 (66.5%)	1.18 (0.61 - 2.28)	0.627
**Primary kidney disease:**					
Diabetic kidney disease	69 (27.2%)	55 (25.7%)	14 (35.0%)	1.82 (0.90 - 3.71)	0.095
Primary glomerular disease	50 (19.7%)	48 (22.4%)	2 (5.0%)	0.22 (0.05 - 0.93)	**0.039**
Interstitial nephropathy	15 (5.9%)	13 (6.1%)	2 (5.0%)	0.77 (0.18 - 3.25)	0.726
Polycystic kidney disease	13 (5.1%)	12 (5.6%)	1 (2.5%)	0.59 (0.08 - 4.35)	0.606
Nephrosclerosis	18 (7.1%)	16 (7.5%)	2 (5.0%)	0.86 (0.20 - 3.61)	0.836
Systemic disease	8 (3.2%)	6 (2.8%)	2 (5.0%)	2.13 (0.51 - 8.94)	0.303
Others or unknown	81 (31.8%)	64 (29.9%)	17 (42.5%)	NA	NA
**Time on KRT (years)**	2.4 (IQR: 1.1-5.3)	2.2 (IQR: 1.1-5.2)	3.3 (IQR: 1.6-5.8)	0.99 (0.97-1.02)[d]	0.624
**Chronic hemodialysis done in a hospital**	120 (47.2%)	104 (48.6%)	16 (40.0%)	0.78 (0.39 - 1.56)	0.487
**Received treatments prior to infection:**					
Angiotensin converting enzyme inhibitors (ACEi)	43 (16.9%)	38 (17.8%)	5 (12.5%)	0.66 (0.26 - 1.70)	0.392
Angiotensin receptor blockers (ARB)	58 (22.8%)	50 (23.4%)	8 (20.0%)	0.89 (0.41 - 1.94)	0.771
ACEi or ARB	99 (39.0%)	86 (40.2%)	13 (32.5%)	0.77 (0.39 - 1.50)	0.443
Non-steroidal anti-inflammatory drugs	11 (4.3%)	9 (4.2%)	2 (5.0%)	1.17 (0.28 - 4.86)	0.831
**Clinical presentation:**					
Asymptomatic	44 (17.3%)	42 (19.6%)	2 (5.0%)	0.23 (0.06 - 0.97)	**0.045**
Fever	163 (64.2%)	128 (59.8%)	35 (87.5%)	3.46 (1.35 - 8.84)	**0.009**
Respiratory symptoms[a]	138 (53.3%)	108 (50.5%)	30 (75.0%)	2.29 (1.12 - 4.68)	**0.024**
Dyspnea	72 (28.4%)	48 (22.4%)	24 (60.0%)	3.62 (1.90 - 6.90)	**<0.001**
Gastrointestinal symptoms[b]	42 (16.5%)	36 (16.8%)	6 (15.0%)	0.89 (0.37 - 2.13)	0.797
Pneumonia	131 (51.6%)	93 (43.5%)	38 (95.0%)	17.55 (4.23 - 72.76)	**<0.001**
Lymphopenia	160 (63.0%)	122 (57.0%)	38 (95.0%)	10.81 (2.61 - 44.84)	**0.001**
**Hospitalization**	166 (65.4%)	127 (59.4%)	39 (97.5%)	22.64 (3.11 - 164.84)	**0.002**
**Hospitalization days**	14.2 ± 13.2	14.7 ± 13.5	12.7 ± 12.0	0.89 (0.73 - 1.07)[e]	0.208
**Admission to ICU**	25 (9.8%)	14 (6.5%)	11 (27.5%)	1.78 (0.88 - 3.60)	0.108
**Mechanical ventilation**	27 (10.6%)	11 (5.1%)	16 (40.0%)	4.29 (2.16 - 8.50)	**<0.001**

Table S2. - Treatments received by elderly patients on hemodialysis with COVID-19 and univariate Cox regression analysis. Treatments evaluated in all elderly patients (≥65 years-old) with known outcome and only in hospitalized elderly patients with known outcome. Values expressed in n (%). HR: Hazard ratio and 95% confidence interval for the ratio. ICU: Intensive Care Unit.

	**All elderly patients**				**Hospitalized elderly patients**			
	**Survivors (n = 437)**	**Non-survivors (n = 239)**	**HR (95% CI)**	***p* value**	**Survivors (n = 291)**	**Non-survivors (n = 224)**	**HR (95% CI)**	***p* value**
**Lopinavir/Ritonavir**	116 (26.5%)	80 (33.5%)	1.40 (1.06 - 1.85)	**0.019**	113 (38.8%)	80 (35.7%)	0.98 (0.74 - 1.31)	0.917
**Hydroxychloroquine (HCQ)**	292 (66.8%)	179 (74.9%)	1.69 (1.18 - 2.42)	**0.004**	250 (85.9%)	176 (78.6%)	0.68 (0.46 - 1.00)	0.053
**Interferon beta**	6 (1.4%)	13 (5.4%)	2.54 (1.45 - 4.47)	**<0.001**	6 (2.1%)	13 (5.8%)	1.95 (1.11 - 3.44)	**0.020**
**Tocilizumab**	16 (3.7%)	12 (5.0%)	0.99 (0.55 - 1.77)	0.965	16 (5.5%)	11 (4.9%)	0.71 (0.39 - 1.31)	0.274
**Glucocorticoids**	91 (20.8%)	61 (25.5%)	1.10 (0.81 - 1.48)	0.551	86 (29.6%)	59 (26.3%)	0.77 (0.56 - 1.05)	0.095
**Azithromycin**	159 (36.4%)	74 (31.0%)	0.87 (0.66 - 1.14)	0.302	118 (40.6%)	73 (32.6%)	0.79 (0.60 - 1.04)	0.094
**Combination of HCQ and Azithromycin**	132 (30.2%)	67 (28.0%)	0.95 (0.72 - 1.27)	0.748	109 (37.5%)	66 (29.5%)	0.76 (0.57 - 1.02)	0.066

Table S3. - Hospitalization and mortality in Spanish general population and hemodialysis patients with COVID-19 infection (Frequencies shown in Figure 3). Hospitalization and mortality showed by age range. Values expressed in n (%). Data for the Spanish general population obtained from the Spanish National Epidemiological Surveillance Network between January 31, 2020 and August 27, 2020[1,2]. Hemodialysis data obtained from the Spanish COVID-19 Working group database.

GENERAL POPULATION

	**Total cases**	**Hospitalized**	**Mortality**
**<30 years**	85452	2925 (3.42%)	36 (0.04%)
**30-39 years**	57030	4625 (8.11%)	67 (0.12%)
**40-49 years**	67970	9817 (14.44%)	228 (0.34%)
**50-59 years**	67830	15446 (22.77%)	689 (1.02%)
**60-69 years**	49092	18659 (38.01%)	1885 (3.84%)
**70-79 years**	41839	22085 (52.79%)	5011 (11.98%)
**≥80 years**	69256	27307 (39.43%)	13360 (19.29%)
**Total**	**438469**	**100864 (23.00%)**	**21276 (4.85%)**

HAEMODIALYSIS POPULATION

	**Total cases**	**Hospitalized**	**Mortality**
**<30 years**	11	4 (36.36%)	1 (9.09%)
**30-39 years**	17	10 (58.82%)	2 (11.76%)
**40-49 years**	41	21 (51.22%)	2 (4.88%)
**50-59 years**	106	71 (66.98%)	20 (18.87%)
**60-69 years**	185	141 (76.22%)	38 (20.54%)
**70-79 years**	272	217 (79.78%)	95 (34.93%)
**≥80 years**	298	217 (72.82%)	121 (40.60%)
**Total**	**930**	**681 (73.23%)**	**279 (30.00%)**

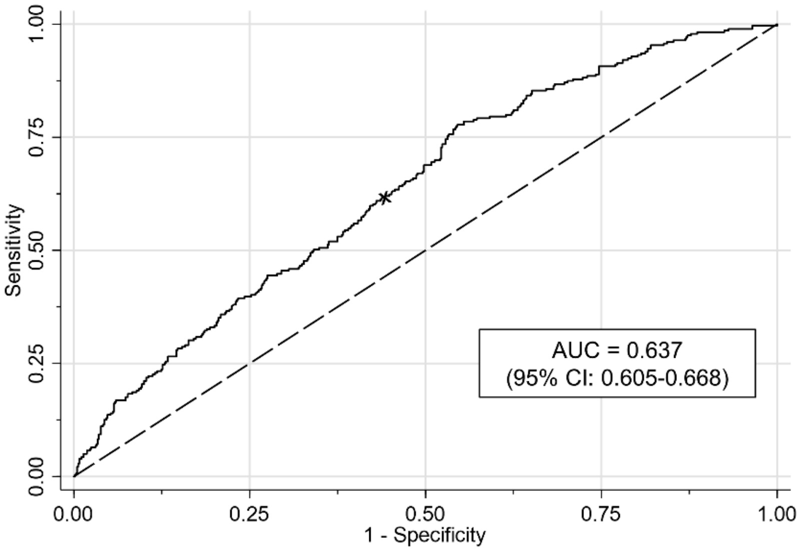

Figure S1. - ROC curve depicting age as a predictor of mortality in elderly haemodialysis patients affected by COVID-19. Age alone is a significant predictor of mortality with an AUC 0.637. The X indicates the age cut-off value (74.8 years) with the best sensitivity and specificity. This value has an efficiency of 58.2% for correctly classifying survivors and non-survivors.

MEMBERS OF THE SPANISH COVID-19 CKD WORKING GROUP

Ainoa Hernando, Alejandro Pérez, Alfonso Cubas, Alfonso Iglesias Alfonso Pobes, Amir Shabaka, Amparo Bernat, Ana Roca, Ana Beatriz Muñoz Díaz, Ana Isabel Martínez, Anabertha Narváez, Andrés Villegas, Angel M Sevillano, Aniana Oliet, Anna Gallardo, Anna Patrícia Balius, Antía López, Antoni Bordils, Antonio Crespo, Antonio Gascón, Antonio Pérez, Argimiro Gándara, Armando Coca, Auxiliadora Mazuecos, Beatriz Diez, Beatriz María Durá, Begoña Rincón, Belén Gómez, Belén Moragrega, Boris Gonzales, Bruna Natacha Leite, Carlos Antonio Soto, Carlos Jarava, Carlos Jesús Cabezas, Carlos Jimenez, Carmen Cabré, Cassandra Emma Puig, Cecilia Dall'Anese, Celestino Piñera, Charo Llópez, Claudio José Horno, Concepción Álamo, Cristina Jimeno Griñó, Cristina Lucas, Cristina Medrano, Daniel Eduardo Villa, Diana Manzano, Diego Rodríguez-Puyol, Dioné González, Eduardo Muñoz de Bustillo, Elena Araceli Jiménez, Elena Calvo, Elena Castillón, Elena Giménez, Elena Gutiérrez, Elena Vaquero, Elvira Esquivias, Emilio Sanchez, Emma Huarte, Enriqueta González, Ernesto Francisco Valga, Eva Alvarez, Felipe Sarró, Felisa Martínez, Fernando Fernandez, Fernando Henriquez, Fernando Simal, Fernando Tornero, Francisco Roca, Francisco Javier Centellas, Francisco Llamas, Gabriel Ledesma Sánchez, Gema Velasco, Gracia Mª Alvarez, Greissi Jeniree Garcia, Gustavo Useche, Hernando Trujillo, Ignacio Cidraque, Ignacio lopez, Inés Aragoncillo, Iñigo Moina, Isabel Berdud, Isabel Garcia, Isabel María Saura, Iván Chamorro, Jaime Sanz, Jary Perello, Javier Arrieta, Javier Vian, Jessica Isabel Urdaneta, Jesus Calviño, Jesús Grande, Jesus Martin, Joan Manuel Gascó, Joaquín de Juan, Joaquin Manrique, Jorge Reichert, José Antonio Herrero, José Jesús Broseta, José Luis Pérez, Juan Carlos Martínez, Juan Casa, Juan Cristobal Santacruz, Juan Manuel Buades, Juan Villaro, Laura Álvarez, Laura Espinosa, Laura Llinàs, Laura Muñiz, Laura Sánchez, Laureano Perez, Lien Winderickx, Liliana Morán, Lucía Sobrino, Luis Alberto Blázquez, Luis Sánchez Cámara, Luis Fernando Domínguez, Luis Gil, Mª Dolores Prados, Mª Jesús Moyano, Mª José Fernández-Reyes, Mª Teresa Naya, Mª Victoria Guijarro, Manuel Heras Benito, Manuel Ramos Díaz, Maria Crucio, María Antonia Fernández, Maria Begoña Aurrekoetxea, Maria del carmen Ruiz, María del Mar Rodríguez, María Esperanza Moral, María Eugenia Palacios, María Isabel Martínez, Maria Jesus Castro, Maria Jimenez, María Luisa Navarro, Maria Noel Martina, María Pérez-Fernández, Maria Pilar Perez, Maria Sanchez-Sanchez, María Teresa Rodrigo, María Teresa García, Maria Victoria Martin, Mariana garbiras, Marta Albalate, Marta Crespo, Marta Luzon Alonso, Marta Sánchez, Martín Giorgi, Mercedes Gonzalez, Miguel angel Suarez, Miguel Rodeles, Milagros Fernandez, Miquel Blasco, Mª Dolores Arenas, Montserrat Belart, Montserrat Picazo, Natalia Blanco, Nuria García-Fernández, Orlando Siveri, Oscar García, Oscar Rolando Durón, Pablo Castro, Patricia de Sequera, Paula Munguía, Pedro Abáigar, R Alvarez, Rafael Garcia-Maset, Ramón Jesús Devesa, Ramón López-Menchero, Raquel Diaz, Raquel Ojeda, Raúl Edilberto Alvarado, Raúl García, Roberto Holgado, Rocio Echarri, Rodrigo Avellaneda, Rosa Garcia, Rosa Maria Ruiz-Calero, Rosa Sanchez, Sagrario García, Sandra Castellano, Sara Huertas, Sergio García-Marcos, Sheila Cabello, Silvia Benito, Silvia González, Silvia Moreno, Silvia Ros, Teresa Bada, Teresa Cordal, Vicente Paraíso, Victoria Oviedo, Virginia López de la Manzanara, Yanina García-Marcote, Zakariae Koraichi Rabie

REFERENCES

1. Red Nacional de Vigilancia Epidemiológica. *Informe COVID-19 Nº33.*; 2020. https://www.isciii.es/QueHacemos/Servicios/VigilanciaSaludPublicaRENAVE/EnfermedadesTransmisibles/Documents/INFORMES/Informes COVID-19/Informe nº 33. Análisis de los casos de COVID-19 hasta el 10 de mayo en España a 29 de mayo de 2020.pdf
2. Red Nacional de Vigilancia Epidemiológica. *Informe COVID-19 Nº41.*; 2020. https://www.isciii.es/QueHacemos/Servicios/VigilanciaSaludPublicaRENAVE/EnfermedadesTransmisibles/Documents/INFORMES/Informes COVID-19/Informe COVID-19. Nº 42_03 de septiembre de 2020.pdf
